# Catalysis Sans Catalyst Loss: The Origins of Prolonged Stability of Graphene–Metal–Graphene Sandwich Architecture for Oxygen Reduction Reactions

**DOI:** 10.1002/advs.202304616

**Published:** 2023-10-20

**Authors:** Ali Abdelhafiz, Ji Il Choi, Bote Zhao, Jinwon Cho, Yong Ding, Luke Soule, Seung Soon Jang, Meilin Liu, Faisal M. Alamgir

**Affiliations:** ^1^ Department of Nuclear Science and Engineering Massachusetts Institute of Technology 77 Mass Ave Cambridge, MA 02139 USA; ^2^ School of Materials Science and Engineering Georgia Institute of Technology 771 Ferst Drive Atlanta GA 30332 USA

**Keywords:** catalyst degradation mechanism, hybrid catalyst, oxygen reduction reaction, proton exchange membrane fuel cell (PEMFC), graphene

## Abstract

Over the past decades, the design of active catalysts has been the subject of intense research efforts. However, there has been significantly less deliberate emphasis on rationally designing a catalyst system with a prolonged stability. A major obstacle comes from the ambiguity behind how catalyst degrades. Several degradation mechanisms are proposed in literature,   but with a lack of systematic studies, the causal relations between degradation and those proposed mechanisms remain ambiguous. Here, a systematic study of a catalyst system comprising of small particles and single atoms of Pt sandwiched between graphene layers, GR/Pt/GR, is studied to  unravel the degradation mechanism of the studied electrocatalyst for oxygen reduction reaction(ORR). Catalyst suffers from atomic dissolution under ORR harsh acidic and oxidizing operation voltages. Single atoms trapped in point defects within the top graphene layer on their way hopping through toward the surface of GR/Pt/GR architecture. Trapping mechanism renders individual Pt atoms as single atom catalyst sites catalyzing ORR for thousands of cycles before washed away in the electrolyte. The GR/Pt/GR catalysts also compare favorably to state‐of‐the‐art commercial Pt/C catalysts and demonstrates a rational design of a hybrid nanoarchitecture with a prolonged stability for thousands of operation cycles.

## Graphical abstract

Catalyst protection through sandwiching between two graphene sheets enhances stability without compromising the activity to catalyze oxygen reduction reaction in acidic medium. Spectroscopic and computational analyses reveal the catalyst degradation mechanism. Under oxygen reduction reaction (ORR) conditions, evolution of point defects in graphene top layers provides a tortuous path for single atoms to reach the catalyst surface to catalyze ORR.

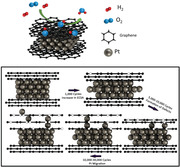

## 1 Introduction

Proton exchange membrane fuel cell (PEMFC) has the oxygen reduction reaction (ORR) as the key electrochemical reaction at the cathode side. Three objectives when developing new electrocatalysts either for ORR or other electrochemical reactions must be well optimized: enhancing the catalytic activity, reducing the cost of the catalyst, and increasing its lifetime. In general, over three decades scientists have been paying significant attention to design alternative electrocatalysts with higher activity and reduced cost. Numerous efforts have been devoted, when designing a new ORR electrocatalyst, to increasing its activity through strain engineering, and reducing the cost by replacing the expensive Pt component with other earth abundant transition metals. A prominent strategy is alloying of Pt with other secondary metals (e.g., Co,^[^ ^1^, ^2^, ^3^ ^]^ Pd,^[^ ^4^, ^5^, ^6^ ^]^ or Ni^[^ ^7^, ^8^ ^]^). Another strategy is forming a core@shell^[^ ^9^, ^10^, ^11^ ^]^ architecture where Pt is the shell layer and secondary metals act as a core support. Through crystal mismatch, mechanical strain induced on Pt shell enhances its activity. Nevertheless, these attempts fail in transcending the activity‐stability dilemma: the higher activity achieved often degrades the stability. Transition metals (e.g., Ni, cu, or Fe) are less stable under harsh acidic environment and highly oxidizing potential, under which PEMFC operates. Different degradation mechanisms have been proposed in literature, such as catalyst atoms dissolution, particle detachment from the support, Ostwald ripening, particles agglomeration, or catalyst‐support corrosion.^[^ ^12^, ^13^, ^14^, ^15^ ^]^ Those mechanisms could happen simultaneously with different kinetics according to the catalyst design.

Evaluating the catalytic activity has very rigorous metrics and is the main point of focus in all the presented studies in literature. Catalyst degradation is equally, if not significantly more, important problem to understand. Nevertheless, assessing the instability/degradation of an electrocatalyst system does not receive the same level of diligence. While significant research efforts are devoted to understanding the origin of activity enhancement, a negligible amount of effort, by contrast, is dedicated to understanding the core mechanisms of activity degradation and providing a robust solution to resolve them.^[^ ^16^ ^]^ We believe, given this substantial gap in literature, that providing a versatile electrocatalytic system which possesses high activity without noticeable activity degradation over prolonged usage, is critically important to the catalysis community.

We hypothesized that a strategy to mediate or resolve the multiple degradation mechanisms simultaneously could be achieved by imposing physical constraints on the catalyst, by supporting from above and below by a mechanically robust material. This support material should also not be a significant hindrance to the surface chemical required of the catalyst. This requisite “chemical transparency” has been demonstrated earlier by Abdelhafiz and Alamgir^[^ ^17^ ^]^ to be possible using single‐layer graphene covering Pt catalysts. Graphene, with its mechanical robustness and chemical stability, has shown great promise to replace conventional carbon black catalyst supports.^[^ ^18^, ^19^, ^20^ ^]^ Recent efforts provided direct conceptual proof of utilizing single layer graphene as Pt catalyst support for ORR, Pt/GR, and further demonstrated enhanced catalyst activity resulting from a graphene‐imposed compressive strain on catalyst atoms.^[^ ^19^, ^20^, ^21^, ^22^ ^]^ Interestingly, on curved sp^2^ carbon surfaces, such as carbon nanotubes, Pt deposits as particles but does so with strong interaction with the C atoms that force the Pt into anisotropic strain.^[^ ^23^ ^]^ The epitaxial relations in the Pt/GR structure ensures that properties emanating from structural anisotropies of the graphene are imprinted onto the surface Pt layer. This is seen in the different molecular chemisorption energies onto Pt that follow the zigzag versus the armchair directions of the underlying graphene.^[^ ^24^ ^]^ One can imagine similar transfer of tuned work‐function of catalyst support through the graphene conduit onto the catalyst overlayers.^[^ ^25^ ^]^

Ripening/agglomeration of Pt catalysts is prevented by the epitaxial relation between graphene and Pt^[^ ^20^, ^26^, ^27^ ^]^ and, when graphene was used as a protective cap over the catalyst, resulted in unprecedented increase in the catalyst lifetime.^[^ ^17^ ^]^ By forming a hybrid catalyst with Pt atoms underneath a single layer of graphene can transcend limitations on catalyst lifetime with no penalty to ORR activity. Two obstacles remain toward realizing the proofs‐of‐concepts Pt/GR and GR/Pt/support architectures into real catalysts. First, it is not known if the effects would persist if combined into a GR/Pt/GR/support sandwich architecture. Second, if multilayer graphene can replace its single‐layer counterpart, then the catalyst synthesis would be more tractable. Of course, for this latter case, the fundamental relationship between activity/stability and the number of sandwiching graphene layers would first have to be established.

In this work, we report a highly robust and efficient ORR electrocatalyst in a unique sandwich structure with mixture of single atomically dispersed and Pt nanoparticles catalysts between two sets of graphene sheets (i.e., support and cap). The effect of graphene cap thickness (the number of layers) on catalyst stability and electrocatalytic activity for ORR has been systemically studied. A combination of experimental and computational analysis presented to unravel the degradation mechanism of electrochemical active surface area (ECSA) and identify the origins of electrocatalytic activity enhancement. Capping metal catalyst with three‐layer graphene thick sheet showed optimal performance with transcending catalyst stability over 20K durability testing without sacrificing ORR activity. The synthesized architecture is flexible and can be extended to catalyze a wide variety of electrochemical reactions, where stability is maximized, without compromising the catalytic activity.

## 2 Results and Discussion

**Figure 1:**
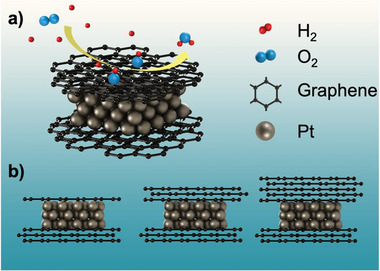
a) Schematic illustration of ORR at graphene/Pt/graphene sandwich structure. b) Sample sets studied with different graphene thicknesses (one, three, and five layers) acting as a cap covering Pt‐nanoparticles.

Three sample sets synthesized with Pt small nanoparticles sandwiched between two sets of graphene sheets (GR/Pt/GR), as shown in **Figure** **1**. Bottom sheet (i.e., support) is three‐layer thick graphene for all cases studied herein, while top sheet (i.e., protective cap) varied from one‐, three‐ to five‐layer thick graphene, each represents a sample set. **Table** **1** gives a notation for each sample set, as will be referred to in the presented thesis hereon. Further details about the experimental procedure can be found in the Supporting Information.

**Table 1:** Graphene‐Pt‐graphene samples’ notations and their respective composition.

Notation	Graphene support layer number	Graphene cap layer number
GR1/Pt/GR	3	1
GR3/Pt/GR	3	3
GR5/Pt/GR	3	5

### 2.1 Structural Analysis and Corresponding Electrocatalytic Activity

**Figure 2:**
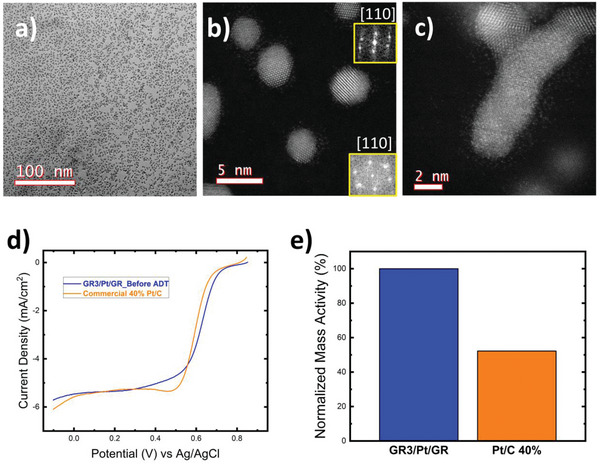
a) TEM image shows GR/Pt/GR sandwich structure with a high monodispersity and random coverage of Pt nanoparticles on graphene. b) STEM image of Pt spherical nanoparticles. Insets are the corresponding FFT patterns along <110> zone axis. c) STEM image of an individual Pt multipod with single atomically dispersed Pt atoms forming a halo surrounding Pt‐nanoparticle. d) ORR activity comparison of GR3/Pt/GR and state of the art Pt‐C 40% commercial catalysts tested in 0.1 m HClO_4_. Current density depicted through linear sweep voltammetry of GR3/Pt/GR compared to commercial Pt/C 40% before ADT. e) Normalized mass activity bar‐chart comparing current density at 0.9 V versus Reversible hydrogen electrode (RHE) obtained from Linear sweep voltammetry (LSV). Figure of merit was compared by fixing GR3/Pt/GR performance to unity (i.e., 100%) and normalizing Pt/C 40% performance accordingly at the same metrics.

GR/Pt/GR sandwich structure was investigated using high resolution transmission electron microscopy and scanning transmission electron microscopy (STEM) with an accelerating voltage 300 and 200 KeV, respectively, to interrogate the morphology of Pt structures and their dispersion on 2D graphene films. Sputtering power and deposition time were varied incrementally to monitor the growth mode of Pt nanoparticles on graphene. Detailed experimental setup and synthesis optimization can be found in the Supporting Information. A set of samples was sputtered for 6, 15, 26, and 60 s at 3 W power. **Figure** **2**a shows high monodispersion of spherically shaped Pt nanoparticles deposited on stand‐alone graphene sheet. Fast Fourier transform (FFT) analysis of STEM images showed that Pt nanoparticles grew along {110} crystal orientation, as shown in Figure 2b, which is known to possess higher electrocatalytic activity for ORR compared to other Pt crystal orientations, as studied by Norskov et al.^[^ ^6^, ^7^ ^]^ The interplanar distance deduced from FFT (inset Figure 2b) of Pt adatoms showed a compressive strain with shorter Pt─Pt bond distance of 2.5–2.6 Å instead of the unstrained 2.78 Å expected in bulk Pt. The induced compressive strain on Pt adatoms have shown an effect toward enhancement of ORR activity.^[^ ^8^, ^9^, ^10^, ^11^, ^12^, ^13^, ^14^ ^]^ In addition, multipods interconnects (Figure 2c) were observed with polycrystalline structure with growth orientation along {100} direction. The observation of compressed Pt─Pt lattice parameter and the existence of less energetically favorable <100> Pt crystal orientation, align with our previous observation where Pt adatoms epitaxially growing on graphene. Hence, graphene/Pt initiate an intimate interaction as a hybrid catalyst, where both catalytic activity and stability are tremendously enhanced. STEM analysis of GR/Pt/GR sandwich structure showed a very interesting observation consistently, where all Pt nanoparticles, regardless of their morphologies, showed a halo of individual Pt atoms surrounding particles’ periphery. This observation implies the high stability of GR/Pt/GR sandwich structure. Samples were subjected to 300 and 200 KV for 2 h each, with 10 d apart; however, single atomically dispersed Pt halo remained intact. This observation is indicating the existence of a mixed single atomically dispersed (single atom catalyst, SAC) and Pt nanoparticles, sandwiched between two graphene sheets. This should contribute to increasing the density of catalytically active sites and have significant influence on the electrochemical performance of GR/Pt/GR sandwich structure, as will be discussed in the following sections.

Two desired objectives for synthesized commercially competitive catalyst are prolonged stability and increased activity. Catalytic activity of GR/Pt/GR sandwich structure was evaluated toward ORR. ORR catalytic activity was tested in an oxygen saturated 0.1 m HClO_4_ with a negative going sweep (0.9: −0.1 V vs Ag/AgCl) at 10 mV s^−1^ scan rate. Different graphene top layer (i.e., cap) was used (one, three, and five layers thick) to demonstrate the effect of graphene thickness coating on ORR activity, and in the following section we are establishing the same comparison in regard of catalyst stability. Samples with five‐layer thick graphene (i.e., GR5/Pt/GR) showed no ORR activity. On the other hand, samples with single layer graphene (i.e., GR1/Pt/GR) showed clear activity toward ORR, which agrees with other literature reports confirming ORR activity for similar case scenario.^[^ ^17^ ^]^ Surprisingly, GR3/Pt/GR sandwich structure with three‐layer thick graphene showed noticeable catalytic aptitude toward ORR. Mass activity of sandwich structure outperformed that of state‐of‐the‐art commercial Pt/carbon catalyst counterpart. As a universal metric to evaluate ORR mass activity, current density was reported at 0.9 V versus RHE reference electrode. GR3/Pt/GR sandwich structure showed twofold enhancement in mass activity and 35 mV less overpotential at half‐wave of LSV, compared to state‐of‐the‐art commercial Pt/carbon catalyst, as shown in Figure 2d,e.

GR3/Pt/GR activity enhancement is believed to originate from the intimate contact between Pt and graphene, where Pt─Pt is affected by templated growth on graphene.^[^ ^17^, ^19^ ^]^ FFT and image analysis of TEM images showed contraction of Pt─Pt bond distance with ≈4%, observed earlier for Pt grown on graphene when compressive strain was induced on Pt adatoms.^[^ ^19^ ^]^ This was reflected with an increase in GR3/Pt/GR intrinsic activity compared to state‐of‐the‐art Pt/C catalyst. Pt metal is at the strong Pt‐oxygen interaction side of the volcano plot.^[^ ^28^, ^29^, ^30^ ^]^ To enhance ORR activity, oxygen bonding to Pt should be weakened to facilitate the desorption of ORR reaction intermediates. Compressive strain affects metal adatoms d‐band center position, pushing d‐band center down (further away) from the Fermi level.^[^ ^11^, ^29^, ^30^, ^31^, ^32^ ^]^ Thus, antibonding states of metal adatoms are more filled where interaction with reaction intermediates (i.e., oxygen or hydroxyl) becomes weaker. In addition, the synthesized mixed single atomically dispersed and small nanoparticles promoted higher precious metal catalyst (i.e., Pt) utilization with larger surface area exposed per mass loading of Pt. However, stability of nonchemically bonded single Pt atoms was shown to be significantly weaker compared to uniform wetted Pt layers on graphene (i.e., 2D Pt sheets).^[^ ^22^ ^]^

### 2.2 Electrochemical Stability Testing

ECSA was tracked through observing changes of hydrogen waves (H_upd_) peaks’ area (0.0: −0.2 V vs Ag/AgCl as a ref electrode).^[^ ^33^, ^34^, ^35^ ^]^ Electrochemical testing was performed in a nitrogen saturated 0.1 m HClO_4_. Cyclic voltammetry (CV) characterization sweeps were obtained at a rate of 50 mV s^−1^ from −0.2: 1.0 V versus Ag/AgCl. Direct comparison between state‐of‐the‐art commercial Pt/carbon (HISPEC4000) and GR/Pt/GR sandwich structures showed superior catalyst stability for our sandwich structure for all graphene‐capped samples regardless of their thickness. Accelerated durability testing (ADT) results showed good stability for GR1/Pt/GR sandwich structure, surviving ≈78% of ECSA after 5K cycles; however, ECSA suffered from rapid ECSA loss in the subsequent cycles up to 15K cycles. This is plausible due to presence of defects within graphene, as will be discussed in detail with an aid of an ex situ Raman analysis.

ADT results showed tremendous stability of GR3/Pt/GR sandwich structure with ECSA increasing by 5% and 7% during first 1K and 5K testing cycles, respectively. Sandwich structure with three‐layer graphene survived 84% and 75% of original ECSA after 15 and 20K ADT cycles. This result is a key feature toward establishing state of the art catalyst platforms for oxygen reduction reaction application, where catalyst lifetime is prolonged. Sandwich structure with five‐layer graphene thick (GR5/Pt/GR) showed an interesting, yet different, behavior from other sandwich structures counterparts (i.e., GR1/Pt/GR and GR3/Pt/GR) during ADT cycles. Through CV scans before ADT, no H_upd_ (−0.2: 0.0 V vs Ag/AgCl) was observed, similarly no observation of Pt─O reduction peak (0.4: 0.8 V) were cited, however, X‐ray photoelectron spectroscopy (XPS) analysis confirmed Pt presence within the surface vicinity. Increasing ADT cycles showed pronounced Pt─O reduction peak around 0.65 V, with monotonic signal enhancement during ADT cycling up to 30K cycles. Simultaneously, no H_upd_ was observed during ADT cycling up to 30K cycles. Meanwhile, pyrolytic graphene defect's peak was observed during positive CV sweeps at 0.9 V, indicating continuous increase of defect's density during ADT.

**Figure 3:**
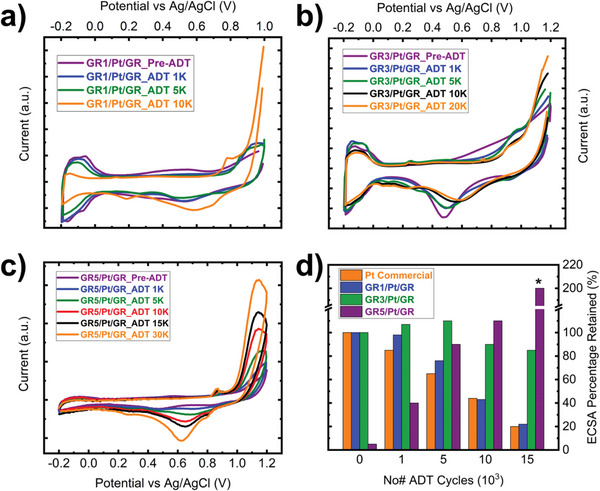
a–c) Cyclic voltammetry curves of GR/Pt/GR sandwich structures with different graphene cap thicknesses: a) 1, b) 3, and c) 5, respectively. d) Bar‐chart of ECSA percent changes with ADT cycling, showing the effect of different graphene thicknesses on electrochemical stability compared to state‐of‐the‐art Pt/carbon commercial catalyst. ECSA percentage change was tracked through integrating H_upd_ wave (−0.2 :0.0 V vs Ag/AgCl) for Pt/carbon commercial, GR1/Pt/GR and GR3/Pt/GR samples, where ECSA was normalized to 100%, representing the initial ECSA for each sample set, individually. On the other hand, GR5/Pt/GR sample ECSA data were tracked by integrating the peak area of Pt─O reduction (0.65 V vs Ag/AgCl). *Initially, GR5/Pt/GR showed no observable ECSA. However, with ADT cycling, ECSA increased. Percentages representing ECSA of GR5/Pt/GR is normalized to the best performing sample (i.e., GR3/Pt/GR pre‐ADT).

The aforementioned observation is well aligned with literature reports;^[^ ^33^, ^36^, ^37^, ^38^ ^]^ as single atomically dispersed Pt catalyst possesses a fast hydrogen‐spillover rate, eliminating the observation of H_upd_ which is limited to ensemble Pt atomic configurations (e.g., steps, defect or threefold hollow sites).^[^ ^39^ ^]^ Pt nanoparticles sandwiched between two sets of graphene sheets (i.e., top and bottom sheets) were encapsulated by top graphene sheet, which acted as a shield suppressing Pt atoms dissolution in the electrolyte. During ADT, graphene may suffer from rupture due to harsh acidic and oxidizing potential, where point defects were introduced by carbon atoms removal from the graphene structure. Thicker graphene (i.e., three and five‐layer sheets) slow down the severity of ORR, providing more barriers to reach bare‐Pt surface, which better describes the longevity of catalyst lifetime inferred from ADT analysis depicted in **Figure** **3**. Nevertheless, point defects provide a pathway for Pt single atoms to hop through vertically between graphene layers until reaching sandwich's surface, before being washed away in the electrolyte. GR5/Pt/GR showed no observation of H_upd_ up to 30K cycles, which shows graphene robustness as a shield protecting bulk Pt (i.e., nanoclusters) from accessibility of H^+^, where Pt─O reduction peak charge can be attributed only to single atomically dispersed Pt atoms hopping through top layers within the graphene cap. As an agreement with the aforementioned hypothesis of Pt migration within graphene sheets, observed ECSA increase of GR3/Pt/GR sandwich structure up to 5K cycles with 7%, indicates that more single Pt atoms move within the top and bottom graphene sheets (i.e., cap and support) during the electrocatalytic reaction. To validate the proposed hypotheses, ex situ Raman analysis was performed within electrochemical ADT cycles and will be discussed in the following section.

### 2.3 Ex Situ Raman Analysis

Graphenes’ 2D and G peak positions and relative intensity of D versus G peaks were analyzed through Raman spectroscopy. Ex situ Raman analysis was performed during ADT cycles at intervals similar to ECSA analysis reported earlier (i.e., before ADT, 1K, 5K, and 10K). Ex situ Raman analysis showed pronounced redshift of 2D peak for GR1/Pt/GR with increasing ADT cycles. Redshift can be attributed to relaxation of preinduced compressive strain within graphenes’ honeycomb structure.^[^ ^40^, ^41^, ^42^ ^]^ Single layer graphene cap suffered from significant change in graphene's nature after 1K ADT cycles, where IG/I2D increased significantly. Increase of G peak intensity (IG) relative to 2D‐peak intensity (I2D) might be due to subfolding of graphene when defects were introduced within graphene structure. In addition, Raman analysis of 2D‐peak position of GR1/Pt/GR sample after 10K cycles showed a shift of ≈77 cm^−1^ compared to 2D‐peak position before performing ADT. ADT is believed to introduce point defects, which was observed by an increase in D‐peak intensity, relative to G‐peak intensity.^[^ ^40^, ^41^, ^42^ ^]^ Point defect presence within graphene is thoughtful to decompress graphene samples, providing more relaxation for carbon atoms in the vicinity of the defect. This observation was validated by examining C 1s peak position from XPS which shifted toward higher binding energies, in a similar observation to Ganesan et al.^[^ ^43^ ^]^ Similar observation of 2D‐peak position of GR3/Pt/GR sample was depicted, however with narrower Raman shifts (i.e., ≈10 cm^−1^), as shown in Figure S7 in the Supporting Information.

**Figure 4:**
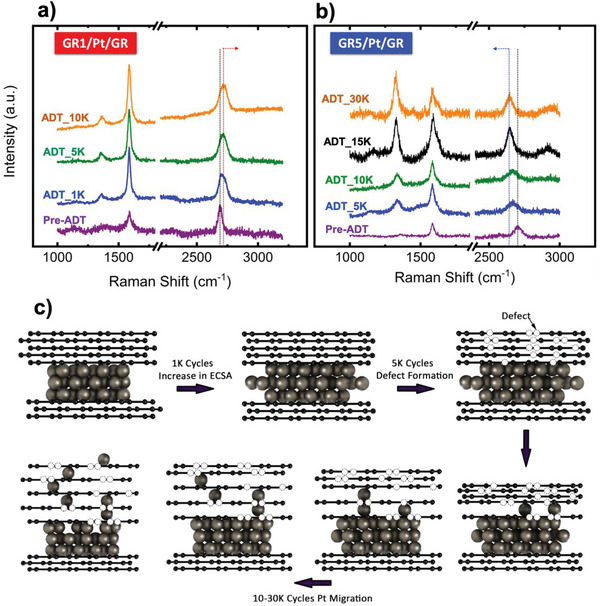
Raman analysis performed during ADT to investigate structural changes in graphene: a) Raman spectra of GR1/Pt/GR sample. b) Raman spectra of GR5/Pt/GR sample. c) proposed degradation mechanism showing Pt single atom diffusion through point defects induced within graphene structure during ADT, resulting in an increase of graphene interlayer spacing (exaggerated preview of interlayer spacing increase to graphically elucidate the proposed mechanism).

GR5/Pt/GR sandwich structure with five‐layer graphene thick showed an opposite trend, where Raman 2D‐peak shifted toward lower Raman shifts (i.e., blueshift) during ADT cycling. GR5/Pt/GR 2D‐peak position was shifted by ≈125 cm^−1^ after 30K ADT cycles. In addition, D‐peak intensity increased significantly after 10K cycles, indicting presence of enormous amount of point defects within graphene's’ honeycomb structure.^[^ ^40^ ^]^ D‐peak intensity increased by a total of approximately fourfold after 30K ADT cycles. Moreover, graphene's’ G peak shape deviated from symmetry due to presence of D’ peak, which indicated disorder of graphene's structure. Blueshift, as described earlier, is attributed to graphene compression, however, point defects density was increased. On the other hand, 2D‐peak position of graphene was reported to be affected by the number of graphene layers. Single layer graphene reported to have 2D‐peak at lower raman shifts compared to thicker graphene sheets, where 2D‐peak position goes through a blueshift systematically with delamination of graphene (i.e., 2D‐peak position order: Monolayer < Bilayer < Trilayer graphene).^[^ ^44^ ^]^ Thus, two possible scenarios can be simultaneously present: first, larger Pt atom, compared to carbon, was squeezed within graphene's defect site inducing localized compressive strain on graphene. Second, single Pt atoms intercalate between graphene layers, which increase the interplanar distances between adjacent graphene layers. Hence, five‐layer graphene behave as pseudo‐delaminated monolayers graphene, as hypothetically suggested in **Figure** **4**c. Therefore, our observation of 2D‐peak blueshift (i.e., to lower raman shifts) with ADT cycles for GR5/Pt/GR can be attributed to increasing the amount of Pt single atoms intercalating/diffusing between graphene sheets.

Raman analysis supports our hypothesis mentioned earlier about the electrochemical performance observed for GR5/Pt/GR with absence of H_upd_ signal.^[^ ^33^ ^]^ As defect density increased, more Pt single atoms were stimulated to hop through graphene. Hence, single Pt atoms trapped within graphene defect site or intercalated between graphene layers at the surface vicinity acted as an active catalytic site for ORR. Concerned about prolonged catalyst durability, GR5/Pt/GR after 30K ADT cycles reserved the relative intensities of IG/I2D peaks, indicating that graphene to a great extent preserved its honeycomb structure regardless of defects presence.^[^ ^40^, ^41^, ^42^ ^]^ This is a plausible demonstration of using graphene as a catalyst‐support and a protective cap, replacing unstable, and easily‐corroded commercial carbon black (e.g., Vulcan XC/72R), which entails boosting catalyst lifetime when tested under harsh acidic conditions.

### 2.4 Computation Analysis of Graphene Defects Generation and Pt Migration

Density functional theory (DFT) was used to investigate the atomic migration of Pt atom in a multiple atom defect site. We used the Vienna Ab initio simulation package (VASP)^[^ ^45^ ^]^ software package, where the electronic interactions and geometric optimization were performed using Perdew–Burke–Erzenhof exchange‐correlation functional within the generalized gradient approximation.^[^ ^46^ ^]^ Atomic interaction energies between atomic species are obtained using a dispersive forces correction method by Grimme,^[^ ^47^ ^]^ and the core and valence electrons are handled by the projector augmented wave method.^[^ ^48^ ^]^ The plane‐wave energy cutoff of 450 eV was adopted, and the Brillouin zone was sampled with 2×2×1 Monkhorst–Pack *k*‐point mesh with spin polarization. Computational models as shown at Figure 1a,b, where the dimension of graphene is selected to have 12.30 Å × 12.30 Å wide to accommodate the defect and Pt clusters.

**Figure 5:**
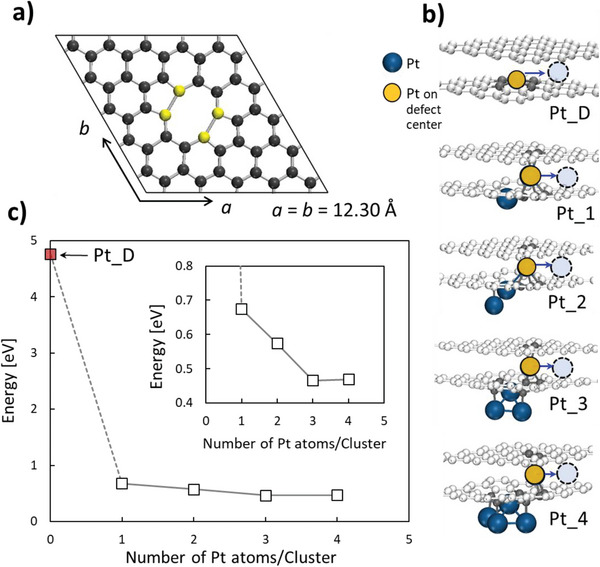
Schematic illustration of a) the computational model of a graphene defect (multiatomic defect is introduced on the graphene) and b) local configurations of Pt clusters formed at the bottom of graphene layers, (Pt_*n*), where *n* indicates the number Pt atoms of each cluster. Dotted circle indicates the position after the Pt migration. Single Pt atom at Pt_0 locates in the middle of defect site forming strong Pt─C covalent bonds, but it deviates from the central binding position upon introducing a Pt cluster. c) Change in the energy barrier for the migration of a Pt atom as a function of the number of Pt atoms in the cluster. The energy barrier becomes constant when *n* ≥ 4.

Various types of defects can be introduced on the graphene during growth or processing,^[^ ^16^ ^]^ affecting electronic and/or mechanical properties of the graphene. According to Raman data presented earlier and literature support,^[^ ^16^ ^]^ point defect is considered to be the dominant defect type to occur in graphene during ADT. Three major possible point defect models have been studied in literature: single vacancy, double vacancy and Stone‐Wales defects. Among them, single and double vacancy defects have very similar formation energy; however, the size of a single vacancy is smaller than a Pt atom and does not allow appreciable through‐defect Pt‐hopping (i.e., penetration) at PEMFC operating conditions. Similarly, Stone‐Wales defect type, however, generated due to bonding reconfiguration, also resembles smaller size of defect for the larger Pt‐atom penetration. Therefore, in this computational study, we use a double vacancy model as an exemplary multiple atomic defects without dangling carbon atom. The model is schematically illustrated in **Figure** **5**a, showing two pentagons in symmetrically mirrored positions due to structural reconstruction. Atomic Pt is introduced on the defect center (Pt_D) and the cluster models are optimized in structures that are presented in Figure 5b. For a single Pt atom binding, model Pt_0 in Figure 5b, a Pt atom occupies the central position of defect forming strong bonds with four carbon atoms, where the bond length is measured around ≈1.96 Å. Pt─C length between C of defect and Pt of Pt clusters is also ranged between 1.96 and 2.07 Å. Our calculations for Pt adatom interaction with graphene is well supported by literature reports for other metal adatoms (e.g., Au or Ni).^[^ ^17^, ^18^ ^]^ Diffusion energy barrier of metal adatoms was reported be significantly lower when the metal adatoms is trapped between two defect‐free graphene sheets compared to the energy required to liberate an adatom trapped within a graphene defect. Pristine graphene with limited amount of defect has low diffusion energy barrier, where atoms separated off Pt nanoparticles can diffuse freely.^[^ ^18^, ^19^, ^20^ ^]^ This better explains the increase of Pt ECSA for GR3/Pt/GR sample with initial 5K ADT cycles, before a critical threshold of defects‐density was reached where Pt adatoms became more prone to get trapped within defect sites.

In the same context, our DFT analysis implies that Pt clustering or agglomeration at a defect site of a graphene layer of multilayer‐thick graphene is less favorable. Figure 5c shows the highest energy variation of 4.76 eV from the model Pt_0 of Figure 5b. It is found that all of active atoms on the defect are strongly interacting with the single Pt atom. As the Pt clusters are introduced, the reaction energy difference linearly decreases up to Pt_3 from 0.67, 0.57, and 0.47 eV for the Pt_1, Pt_2, and Pt_3, respectively. Pt atoms trapped at a top of a graphene defect is prone to diffuse when another Pt atom reaches defect center from the bottom side of the defect (Figure 5b). Hence, top Pt atoms diffuse between graphene layers till they reach another defect site, while the bottom Pt atoms hop toward the upper side of the graphene defect. Similar observation was shown for other metals adatoms to have less energy barrier to migrate vertically from bottom to top site of a defect than lateral diffusion off the defect‐center.^[^ ^18^ ^]^ In addition, our DFT analysis showed that Pt‐atoms clustering at the bottom side of the defect leads to rearrangement of atomic configuration at the defect that shift the Pt atom (Pt_D) to an off‐center position in between graphene layers. This gives an explanation of the expected catalytic degradation mechanism describing Pt migration through the graphene layers. Figure 5c depicts the reaction energy variation (i.e., energy barrier to liberate Pt atoms from defect site) for the Pt migration from the defect site to an adjacent position where the Pt atom binds with sp^2^ carbon atoms of the graphene layers.

**Figure 6:**
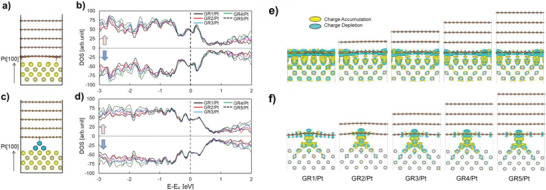
Electronic density of states are calculated for models a) bare graphene‐Pt(100) and (b) shows the DOS as a function of graphene layers. (c) is the computational model of graphene‐Pt cluster on Pt(100) and (d) shows the corresponding DOS features. Charge density differences are calculated for e) graphene/Pt(100) and f) graphene/Pt‐cluster/Pt(100). It is found that direct contact of graphene with the Pt atom/surfaces induces the considerable electronic net charge redistribution at the interface, while additional graphene layers are relatively uninfluenced.

Pristine graphene is fairly inert with respect to chemical reactions because its p_z_ atomic orbitals are strongly coupled and stabilized in a giant, delocalized p bonding system.^[^ ^49^ ^]^ Direct contact of the graphene to the Pt surface, however, will alter the electronic structures at the interface region. By investigating the changes in electronic structure as a function of graphene sheets, we found that the interaction with Pt surface gives negligible effect on the electronic density of states (DOS) of additional graphene layers. First, electron DOS near the Fermi level are investigated as a function of graphene layers on the bare Pt(100) and Pt‐cluster‐on‐Pt (100) surfaces as exhibited at **Figure** **6**a–d, where all systems show nearly identical distribution at the Fermi level regardless of the number of graphene layers. This means that no additional change in Fermi level and electronic structures are made by adding extra graphene layers. Second, even though monolayered graphene on Pt(100) (GR1/Pt) is found to have considerable changes in electronic density distribution on the graphene and Pt surface as presented at Figure 6e,f, the additional graphene layers are found to be uninfluenced by such changes observed in the charge density distribution, indicating that those additional graphene layers are chemically inert. We believe it is due to the relatively large interlayer distance between graphene sheets, therein the electronic structures are not disturbed by the electronic redistribution at the Pt‐Graphene interface. Therefore, while direct Pt‐Graphene interaction is an important key for the generation of chemically active graphene surface, the defects on the graphene layers and the corresponding migration of Pt atoms are a reasonable source of the chemically active graphene surfaces.

### 2.5 GR/Pt/GR Sandwich Activation and Degradation Mechanism

GR/Pt/GR sandwich structure showed interesting electrocatalytic aptitude for ORR with 200% mass activity enhancement compared to state‐of‐the‐art commercial Pt catalyst. The most important aspect of a good electrocatalyst system is a prolonged lifetime. Sandwiched structure showed tremendous stability scaled proportionally with increasing the graphene layers thickness of the top (cap) layer facing the electrolyte. Microscopic imaging showed a consistent phenomenon of single atomically dispersed Pt surrounding the periphery of particles. Stabilizing single atoms requires a strong chemical bond (e.g., ionic or covalent) with underlying support (e.g., carbon). Our graphene does not have extrinsic doping (e.g., N, S, or P) which is usually a defect site to stabilize SACs. Therefore, the strong interaction between the Pt SAC and graphene observed as stabile SACs under TEM imaging conditions, infer a strong chemical intimacy between Pt─C. This phenomenon of covalent‐like bond between Pt and C has been shown by our previous report.^[^ ^17^, ^19^, ^21^, ^22^ ^]^ First, Pt deposition was conducted by very slow sputtering; however, clustering of atoms to form nanoparticles is expected to occur naturally on pristine graphene.^[^ ^50^, ^51^ ^]^ To encapsulate the Pt by the top graphene sheet and make a firm contact, GR/Pt/GR sandwich was baked in air atmosphere for 90 min at 250 °C. The baking step is believed to initiate the migration of single atoms Pt to hop through point defects of graphene toward the surface. Introducing point defects in graphene could happen in oxidizing atmospheres (i.e., air) at 250 °C, while at elevated temperatures (e.g., 400–500 °C) a large number of defects is expected to exist. Therefore, the pathway available for Pt SAC to migrate through toward the surface is the already existing point defects within the graphene. In addition, computational analysis showed that Pt orbitals can only extend through a single layer of graphene, while thicker graphene (two or more layers) screen the accessibility of Pt orbitals to reactions intermediates (H^+^ or O_2_). Therefore, in the case of a five‐layer thick graphene cap (GR5/Pt/GR) no signal was observed for either Pt─O reduction or H_upd_ peaks. As the energy provided during the baking step could only be enough to migrate the Pt atoms only through the bottom‐most two to three layers, but not all the way to the 4th layer at the top of the graphene sheet. On the contrary, samples with three‐layer thick graphene showed both signals during electrochemical CV for Pt─O reduction or H_upd_ peaks, as it is easier for Pt atoms to reach the graphene near the surface. During ADT of GR3/Pt/GR samples set, ECSA was increased monotonically for the first 5K cycles. During that period, and under harsh acidic and oxidizing potential cycling, more point defects can be introduced within the graphene, which facilitate the migration of Pt SAC to be located adjacent the surface, where more active sites are generated. After 10K ADT cycles, the percentage of graphene defects is increased significantly and more Pt can find an easier path to the utmost graphene layer, before being washed away in the electrolyte. GR5/Pt/GR sample set showed a similar phenomenon, where the peak area of Pt─O reduction started to be observed and was monotonically increasing until 30K cycles. On the other hand, no H_upd_ waves was observed which confirms that the signal obtained for Pt was coming from single atoms, not cluster. Single Pt atoms has an extremely rapid hydrogen spill over rate, where the observation of H_upd_ on a single Pt atom is restricted to Pt atoms at the defected sites of bulk particles (i.e., step terraces and kinks).^[^ ^35^, ^52^, ^53^, ^54^, ^55^, ^56^ ^]^

The proposed catalyst activation/degradation mechanism was further supported by Raman analysis. 2D peak of graphene of GR5/Pt/GR was shifted to lower wave numbers, which is an indication on graphene being compressively strained. Pt atoms migrating through the point defects will be entrapped within the graphene defects, until a cluster or few atoms reach underneath to liberate it from the point defect site. This process can be observed to be stochastically challenging, where the ratio of Pt atoms entrapped in a defect site (i.e., time at defect) is much larger than the time spent by other atoms (two or more eventually forming a cluster underneath), which are roaming searching for the same defect site. Pt atoms squeeze the graphene which is inducing compressive strain. Moreover, 2D peak position of graphene is sensitive to the graphene thickness. 2D peak position will shift to lower wave numbers if the graphene thickness is reduced. With Pt clustering under the defect site to surpass the activation energy barrier needed to liberate a Pt atom sitting on top of that graphene defect will start to isolate the adjacent graphene layers from one another. Increasing the percentage of defects during ADT will activate more clusters to occur. Hence, five‐layer graphene thick may behave as stacks of isolated one‐ or two‐layer thick graphene sheets.

Sandwich structure provides a prolonged stability compared to bare catalysts. After 10K ADT cycles, the mass activity of GR3/Pt/GR structure was 660% compared to state‐of‐the‐art Pt catalysts supported on commercial carbon black, as shown in Figure S9 in the Supporting Information. The presented GR/Pt/GR sandwich structure herein demonstrated a versatile structure for amenable for different electrocatalytic reactions other than ORR. Graphene protection mechanism is a revolutionary inexpensive method which can help retain the activity of other functional metallic and semiconductor platforms for applications related to energy generation, environmental, or sensing applications.

## 3 Conclusion

In this work we demonstrate a sandwich structured electrocatalyst with Pt wrapped by two sets of graphene sheets. These GR/Pt/GR sandwich structures showed significant enhancement of catalyst stability in comparison to state‐of‐the‐art commercial Pt/carbon catalyst. The graphene cap worked as a shield preventing the loss of Pt atoms. Optimal thickness of the graphene cap was found to be three‐layer thick, where the graphene and the Pt within this GR3/Pt structure worked in unison as a hybrid catalyst for ORR. At the same time, three‐layer thick graphene showed remarkable stability, surviving approximately five‐times more Pt ECSA compared to state‐of‐the‐art commercial Pt/carbon catalyst. The catalyst degradation mechanism is thought to be through defect formation within the graphene structure, through which Pt atoms migrate toward the surface, before leaching out into the electrolyte. Yet this leaching process is tremendously slowed down by the fact that the graphene defects act as temporary traps for Pt atoms migrating to the surface. The demonstrated GR/Pt/GR sandwich structure provides a versatile material platform that can be tailored not only for electrocatalytic application, but others including environmental, biomedical, and sensing applications. The presented study provides detailed insights unraveling the origin of activity and stability degradation of metal catalysts in acidic medium of oxygen reduction reaction. This is believed to enlighten the way to design functional heterogeneous electrocatalysts with a prolonged stability.

## Conflict of Interest

The authors declare no conflict of interest.

## Author Contributions

A.A. and F.A. conceived the concept. A.A. prepared GR/Pt/GR sandwich structures, performed testing, and characterization of the samples. J.I.C. performed the DFT computational analysis. Y.D. carried out TEM experiments. A.A. analyzed all the data and primarily prepared the paper. J.I.C. and B.Z. helped in paper preparation. S.S.J. supervised DFT computational analysis. M.L. and F.A. supervised the research activities.

## Supporting information

Supporting Information

Supplemental Movie 1

## Data Availability

The data that support the findings of this study are available from the corresponding author upon reasonable request.
