# Supplementary material for: Catalysis Sans Catalyst Loss: The Origins of Prolonged Stability of Graphene–Metal–Graphene Sandwich Architecture for Oxygen Reduction Reactions
Source: Adv Sci (Weinh). 2023 Oct 20;10(34):2304616. doi: 10.1002/advs.202304616 (PMC10700250; doi:10.1002/advs.202304616)
Supplement: Supplementary file 1 — Supporting Information [file ADVS-10-2304616-s001.pdf]

ADVANCED SCIENCE
Open Access

Supporting Information

for *Adv. Sci.*, DOI 10.1002/advs.202304616

Catalysis Sans Catalyst Loss: The Origins of Prolonged Stability of Graphene–Metal–Graphene Sandwich Architecture for Oxygen Reduction Reactions

Ali Abdelhafiz, Ji Il Choi, Bote Zhao, Jinwon Cho, Yong Ding, Luke Soule, Seung Soon Jang*, Meilin Liu* and Faisal M. Alamgir**

Catalysis *sans* catalyst loss: The origins of prolonged stability of graphene-metal-graphene sandwich architecture for oxygen reduction reactions

Ali Abdelhafiz[1,2,*], Ji Il Choi[2], Bote Zhao[2], Jinwon Cho[2], Yong Ding[2], Luke Soule[2], Seung Soon Jang[2,*], Meilin Liu[2,*], Faisal M. Alamgir[2,*]

[1] Department of Nuclear Science and Engineering, Massachusetts Institute of Technology, 77 Mass Ave, Cambridge 02139, United States
[2] School of Materials Science and Engineering, Georgia Institute of Technology, 771 Ferst Drive, Atlanta, Georgia 30332, United States

* Co-corresponding authors

Experimental Section

Copper foil 20 μu thick was cleaned by immersing for 10 min in glacial acetic acid to remove native oxide layer, followed by successive washing under De-Ionized water. CVD chamber is pumped down for 10 minutes, before Cu foil is heated to 1000 °C with a ramping rate of 7 °C/min under a mixture of Hydrogen/Argon (37/63, respectively). Afterwards Cu was annealed at 1000 °C for 30 minutes, before introducing CVD gas mixture ($CH_4/H_2/Ar$). controlling the reaction gases ratios and CVD time enabled fine-tune of graphene thickness (e.g. 1, 3 and 5 layers thick). Finally, temperature is ramped down with an effective rate of 3 °C/min under a stream of H_2/Ar of 2/10, respectively[1-5]. Graphene transfer on gold stubs was performed by etching copper foil in acid bath published elsewhere [6-8].

Pt (99.99%) target was used for sputtering under room temperature on Graphene supported on gold stubs. The sputtering chamber was pumped down to $4e^{-5}$ Torr and kept under vacuum for 1 hour. DC-magnetron sputtering was performed at 3.4 e^{-3} Torr and 3-Watt DC power for 26 seconds, yielding an effective deposition 1 μg/cm^2. Following, graphene capping layer was transferred on top of Pt nanoparticles. Three different graphene-cap thicknesses were used (1, 3 or 5 layers thick), denoted as shown in the following table.

Table S1:Graphene/Pt/Graphene sandwich structure sample annotation

Notation	Graphene support layer number	Graphene cap layer number
GR1/Pt/GR	**3**	**1**
GR3/Pt/GR	**3**	**3**
GR5/Pt/GR	**3**	**5**

Synthesis

Further details about synthesis optimization is presented in the following sections

a) Sputtering deposition rate calibration

Pt sputtering parameters (i.e., pressure, magnetron plasma power, sputtering time, sample distance from sputtering target) were modified to control the deposition rate.

i. Pt was sputtered on glass substrates to calibrate the deposition thickness. at least, three samples were sputtered at the same exact condition, as shown in the table below, to estimate the mass loading. At the beginning of each deposition cycle, throughout the given manuscript, the plasma was striking at the target, however the shatter was not opened for deposition until few minutes to reach a steady state and ensure removing any contaminants that might be accumulated at the target surface. The results showed high consistency of Pt sputtering with 3% variation. The average deposition rate is 10 $\mu g/cm^2 min$ for pressure 8.6 mTorr and power 20 Watt, as shown in table 2.

Table S2:Parameters of initial Pt sputtering trials

**Pressure (bar)**	**Power (Watt)**	**Gas flow rate (sccm)**	**Sputtering Time (min)**	**Weight (mg)**
$8.6E^{-3}$	20	30	60	3.2
			60	3.3
			60	3.2

ii. Further efforts were spent to lower the deposition rate to reach ~1 $\mu g/cm^2$ Pt mass loading, which is equivalent to nominal three atomic layers thick of 2D Pt thin-film. Three atomic layers thick showed, previously, the threshold to observe compressive strain from graphene on Pt adatoms. Compressive strain is crucial to boost ORR activity, as discussed in the given manuscript and as widely presented in literature.

To control the deposition rate series of experiments were performed by varying pressure, sputtering power and distance between sample-stage and sputtering target, as shown in the table below. Testing parameters were specified according to experimental design performed using Minitab software, for a partial factorial design experiment, as shown in table 3

Table S3:Partial factorial design of Pt sputtering parameters

Stage Position	Power (Watt)	Pressure (e^{-3} mbar)	Ar Flow rate (sccm)	Time (min)	Deposition rate (ug/cm^2.min)
0	3	6.3	30	30	4.3
0	5	6.3	30	30	6.8
0	20	6.3	30	30	29
0	5	8.6	30	30	8.4
0	20	8.6	30	20	31
-25	3	3.4	18	30	2.5
-25	3	3.4	18	30	2.6
-25	3	3.4	18	30	2.4
-25	3	3.4	18	30	2.2

iii. Observed deposition rates recorded from the experiments were fed into Minitab software to analyze the significance of each factor. It's can be observed that setting the sample stage further away from the sputtering target (by additional 25 mm, lower than the default position) lowered the deposition rate significantly. Minitab analysis as shown in Figures below showed that data was perfectly fit to a "linear regression model" which will be used to estimate the deposition rate. Random distribution of analyzed data points around Residues (above and below zero dotted line) proves the validity of the model, with unbiased statistically calculated Mean.

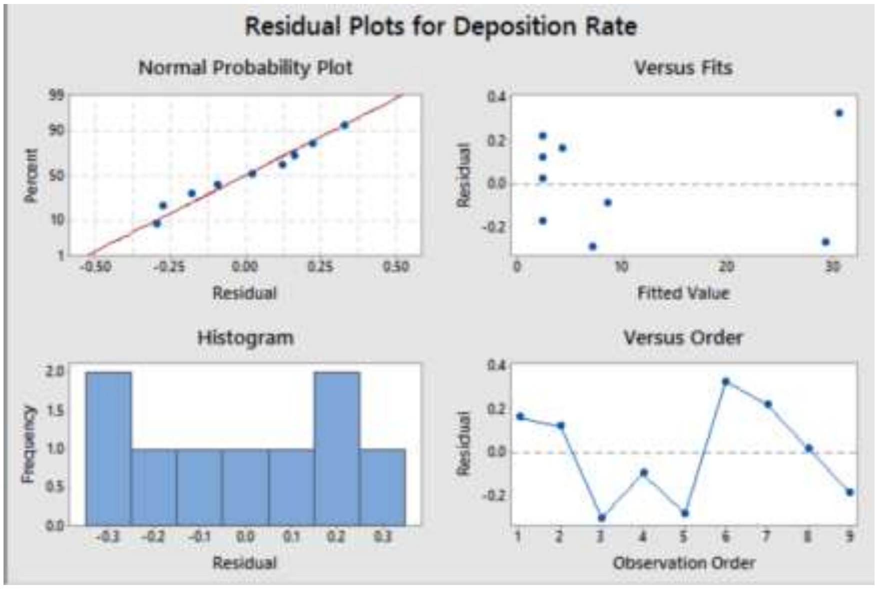

Figure S1: Statistical analysis of the deposition parameters generated by Minitab package

Pareto analysis of predictors showed that both power and pressure have influence on deposition rate, with more superiority given to power. Linear model fits the data very well, which was proven by residuals and variance analyses, as shown in the table below. From the fitted model, the desired deposition of 1 ug/cm^2.min can be achieved at sputtering power of 3 W, pressure 3.4 mTorr and 26 seconds.

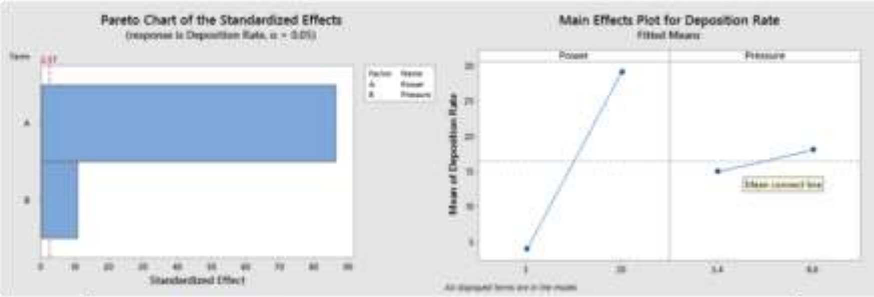

Coefficients

Term	Coef	SE Coef	T-Value	P-Value	VIF
Constant	-4.121	0.250	-16.48	0.000	
power	1.4788	0.0153	96.81	0.000	1.48
pressure	0.6070	0.0506	12.00	0.000	1.48

Regression Equation

Deposition Rate = -4.121 + 1.4788 power + 0.6070 pressure

Figure S2: Estimation of deposition rate based on power and pressure during sputtering analysis

b) Graphene CVD

Graphene synthesis was fabricated in a controlled atmosphere CVD furnace. Cu substrate was first treated with glacial acetic acid for 10 min, before successive rinse with DI water and dry under compressed air flow. Cu foil pieces of 3*9 cm was placed in alumina crucible and introduced into CVD chamber right after. Graphene CVD was typically produced under atmospheric pressure. Ratios of reactive/carrier gases (i.e. CH_4, H_2 and Ar) was controlled through three separate mass flow controllers for each gas line.

c) Fabrication of GR/Pt/GR sandwich structure

i. Graphene is synthesized by CVD process described earlier on Cu foil (i.e. GR/Cu).

ii. Pt deposition was performed on 2*3 cm area of graphene-coated Cu foil (i.e. Pt/GR/Cu).

iii. PMMA resin spin-coated on Pt/GR/Cu for 160 seconds at 3000 rpm. Under room temperature PMMA is left to dry overnight (i.e. PMMA/Pt/GR/Cu).

iv. Cu foil is etched away in acid baths by floating of PMMA/Pt/GR/Cu on top of the solution (as widely published in literature). First, PMMA/Pt/GR/Cu etched in 7.5% HNO3 for 90 seconds, followed by floating on DI-water for 5 min to ensure dilution of any HNO3 remnants. Second, PMMA/Pt/GR/Cu floating on top of ammonium per sulfate solution overnight to remove Cu foil totally (i.e. PMMA/Pt/GR), followed by floating on top of DI-water to remove any acid residues.

v. Floating film of PMMA/Pt/GR is transferred to Au film (i.e. PMMA/Pt/GR/Au), as either:

a. Mirror-polished Au stubs 5*4 mm diameter*thickness respectively, similar to glassy carbon electrode size for RDE measurements
b. Physical Vapor deposited 100 nm Au film on glass substrate (1*1.5 cm) for ECSA and ADT measurements.

vi. PMMA/Pt/GR/Au was heated at 5 °C/min to 250 °C, before soaking for 30 min to ensure strong bonding of GR & Au interface.

vii. PMMA layer was etched away by dipping PMMA/Pt/GR/Au in acetone solvent overnight (i.e. Pt/GR/Au).

viii. Pt/GR/Au either as Au stub or Au fil supported on glass, was coated by second layer of graphene (i.e. cap) similar to steps i-vii, excluding step ii.

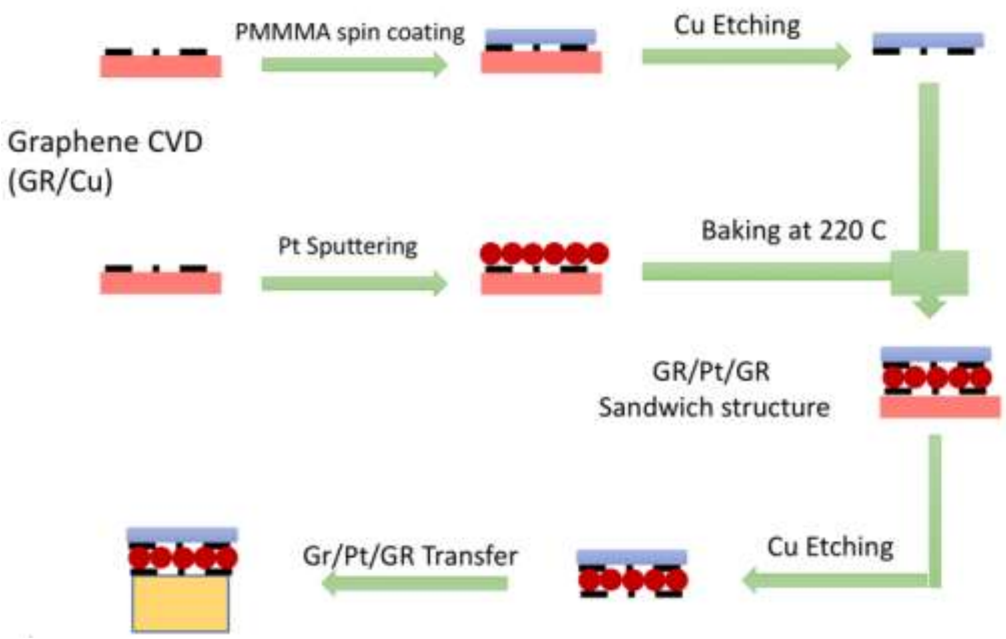

Figure S3: GR/Pt/GR sandwich structure process flow. Started with graphene CVD (dotted black) on copper foil (pink). Followed by either PMMA (blue) coating protection or Pt sputtering (red), before finally both parts were assembled together by heating at 250 °C on gold stubs (yellow)

Electrochemical measurements

Electrochemical measurements were performed in 3-electrode setup with a graphite rod and Ag/AgCl as counter and reference electrodes, respectively. Accelerated durability testing (ADT) and Oxygen Reduction Reaction probing were measured in 0.1M $HClO_4$ electrolyte, saturated with nitrogen and oxygen, respectively. ADT was performed using CV scans 0.4-0.8 V vs Ag/AgCl as a scan rate of 50 mV/s. Changes in ECSA during ADT were investigated by tracking hydrogen adsorption/desorption waves (Hupd) at both positive and negative sweeps at the interval of -0.2:0.0 V vs Ag/AgCl by integrating area under the peak. ECSA was tracked after 1, 5, 10, 15 and 20K cycles. ORR was probed at rotating disk setup (RDE) at 1600 rpm, using liner sweep voltammetry (LSV) 0.9:-0.1V at a scan rate of 10mV/s.

Results

Particle's size analysis was performed using image processing software, with a larger field view

of 150*150 nm. Majority of particle's morphology was either spherical or rod-like shape, while some particles were in a multi-pod interconnects shape. The reported size herein, resembles the critical dimension of the particle's diameter as spheres or rods. A total of 81 particles were measured and results reported as a histogram shown in Figure 1S. Vast majority of particles (41) had a diameter range of 1.6-2.4 nm, followed by 11 particles with diameter 2.4-3.2 nm, 7 particles 3.2-4.0 nm, 5 particles 0.8-1.6 nm and 3 particles of 4.0-4.8 nm in diameter.

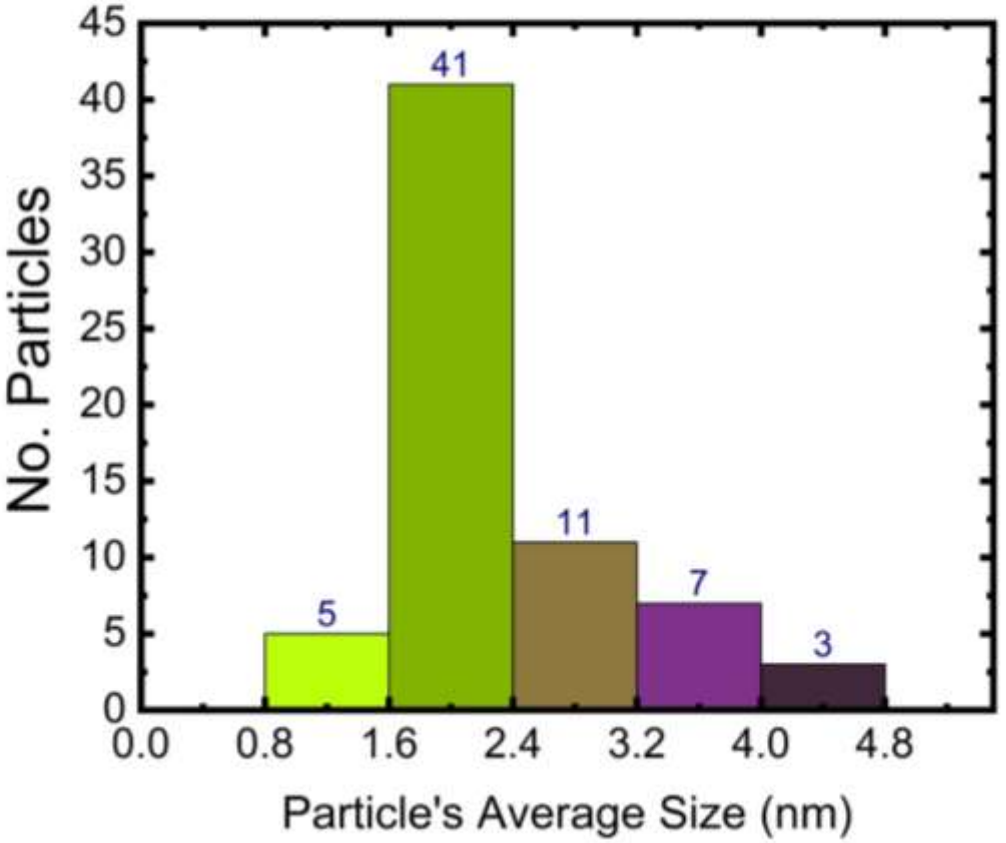

Figure S4: Pt particle's size depicted from TEM analysis over an area of 150*150 nm^2 showing 5 ranges of particle's sizes with 0.8nm each.

Samples fabricated for TEM analysis was prepared similar to the synthesis scheme discussed earlier, however, gold stub was replaced by TEM grids or Si wafers. Addition cross-sectional imaging was conducted. GR/Pt/GR sandwich structure was built on Si wafers. Pieces of 2-3 mm width and 8 mm length was cut out of GR/Pt/GR built on Si wafer. Two pieces were placed face-face, where GR/Pt/GR sides of each piece are facing one another. The two pieces were glued from two opposite sides only (out of four sides for each rectangular Si wafer) to maintain well adhesion. The other two un-glued sides were remained free for electron beam to shine over, at the interface of the two GR/Pt/GR sides of each piece. This experiment was quite difficult, and the imaging resolution was not high enough to yield atomic resolution imaging, however, was an additional conformation of the fabrication of GR/Pt/GR sandwich band.

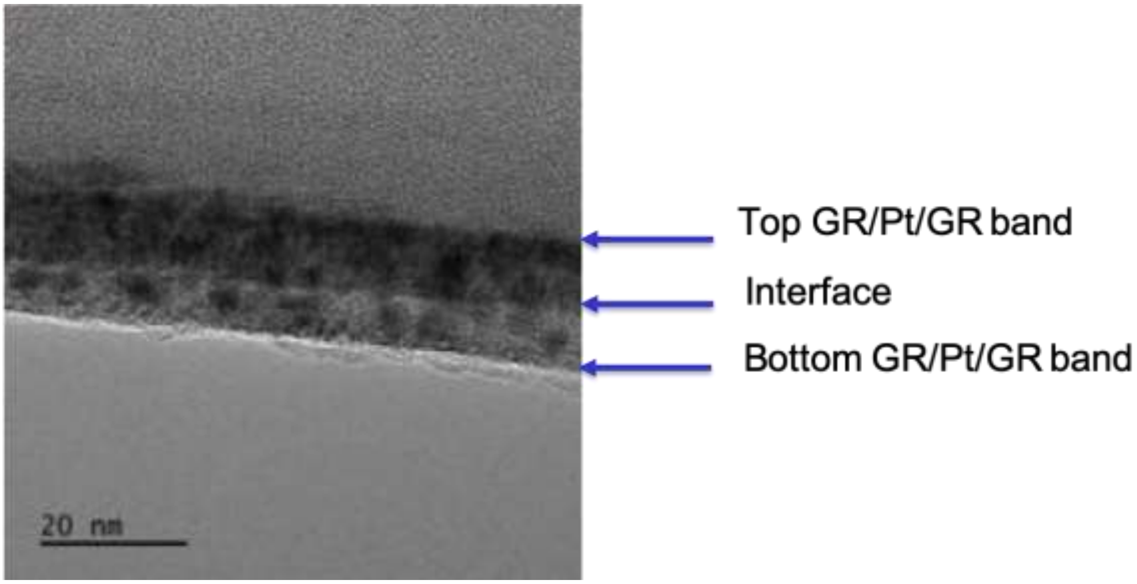

Figure S5: Cross-sectional TEM analysis of GR/Pt/GR sandwich structure. Top and bottom GR/Pt/GR bands were grown on Si wafer pieces, where both bands were placed face-face relative to one another.

XPS was used to confirm the sputtering of Pt nanoparticles, along with its chemical nature. XPS photoemissions of $Pt4f_{7/2}$ and $Pt4f_{5/2}$ showed perfect metallic nature, which is essential to achieved higher ORR activity. In addition, Carbon 1s photoemission of GR/Pt/GR sandwich structure showed a vanishingly small, if any, C=O peak, which is confirming that there is no remnant PMMA protective layer, which was used to handle GR, after acetone etching.

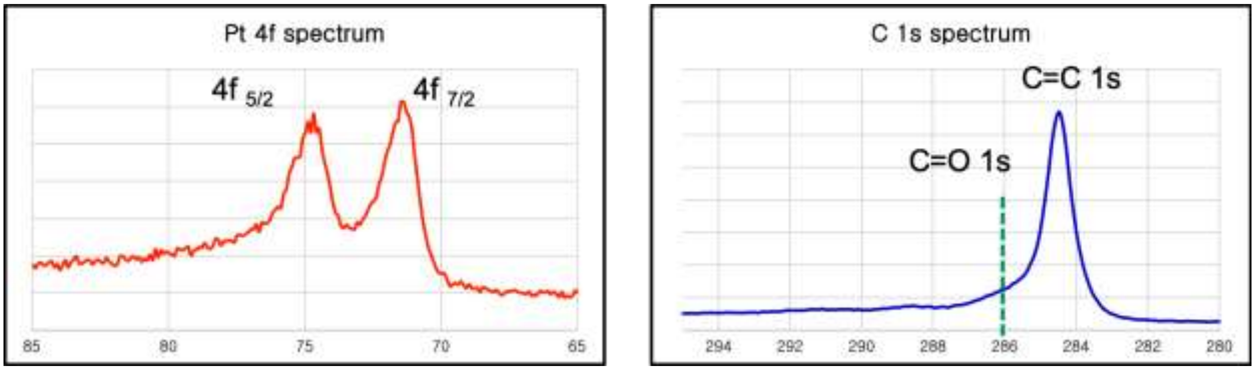

Figure S6: XPS spectra of GR/Pt/GR sandwich structure for high resolution Pt 4f and C 1s photoemissions

Ex-situ Raman analysis was performed using a green laser with a scan speed of 30 mS/cm^{-1}. GR3/Pt/GR sandwich structure capped with 3-layers thick graphene showed slight shift of 2D peak position with ADT cycling to higher Raman shifts, however shifts were less significant compared to GR1/Pt/GR sample set. 2D peak shift was tracking by position of mid-peak position at FWHM.

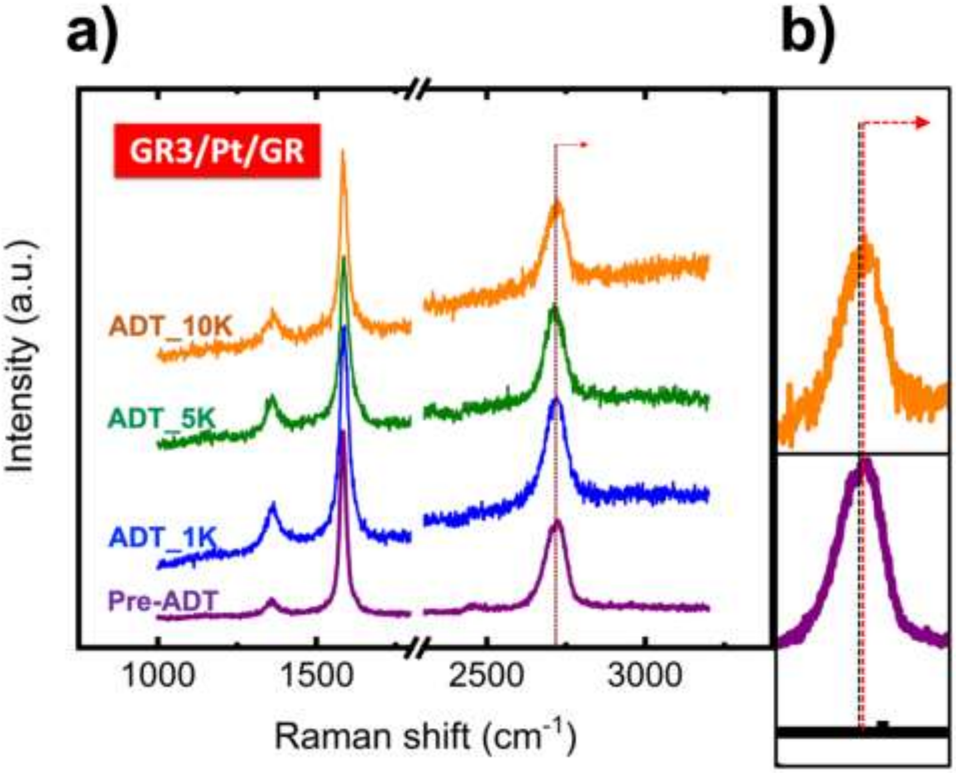

Figure S7: a) Ex-situ Raman spectra of GR3/Pt/GR sandwich capped with 3-layers thick graphene. b) Raman 2D peak zoomed-in view of Pre-ADT and after 10K ADT scans.

Further electrochemical testing and data analysis are presented in the Electrochemical Measurement section at the end of this document. Regarding PEMFC testing, to achieve considerable activity, the lowest possible Pt loading in literature is 100 μg/cm^2 for the cathode side. Our GR/Pt/GR sandwich structure possesses 1 μg/cm^2. Therefore, significant efforts need to be consumed to synthesize the necessary effective Pt area loading, which is an ongoing research effort beyond the presented thesis herein. However, we tried to test the activity of GR3/Pt/GR sandwich structure as a cathode catalyst in a full cell PEMFC stack. Anode catalyst was commercial 40% Pt/carbon with effective loading of 50 μg/cm^2, prepared by hot spray on Nafion-211 membrane. On the other hand, GR3/Pt/GR film was placed physically between Gas Diffusion Layer (GDL Sigracet 39 BC Carbon Paper) and the other side of nafion-211 membrane, and mechanically compressed together. Cell was operated at 65 °C and 100% relative humidity with O_2/H_2 as cathode and node fuels with a flow rate of 300 and 150 sccm, respectively. As a benchmark, Pt/C 40% was used as a cathode catalyst with 100 μg/cm^2 mass loading, while anode catalyst was 50 μg/cm^2. Both anode and cathode catalysts were hand sprayed on the membrane (i.e. Catalyst coated Membrane “CCM” method). Maximum power output was reported to be 301 mW/cm^2 compared to 654 mW/cm^2 for GR3/Pt/GR and Pt/C 40%, respectively, however MEA of Pt/C catalyst was 100 times higher mass loading (i.e. 100 μg/cm^2).

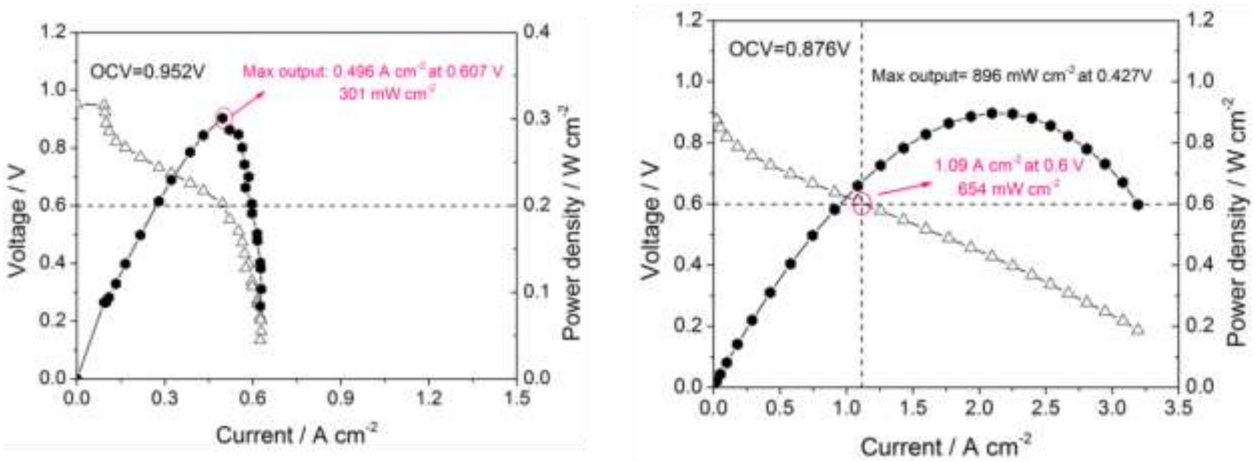

Figure S8: MEA testing in a full stack PEMFC setup under H_2 and O_2 gas flow at anode and cathode side, respectively. Left graph depicts the MEA activity of GR3/Pt/GR as cathode catalyst with 1 μg/cm^2 mass loading. Right graph depicts MEA performance with Pt/C 40% as cathode catalyst with 100 μg/cm^2 mass loading

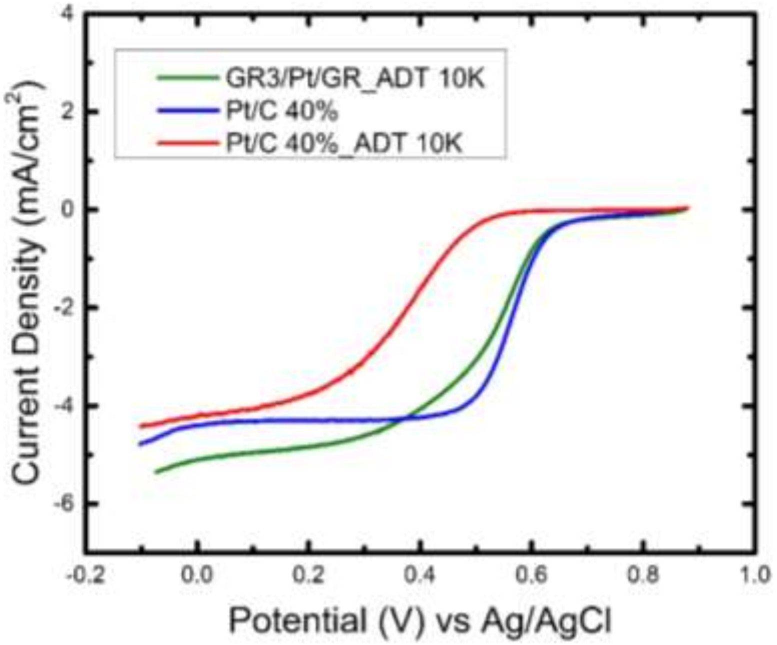

Figure S9: ORR probed through LSV measured in oxygen saturated 0.1 M $HClO_4$. After 10K ADT, GR3/Pt/GR sandwich structure showing comparable activity to Pt/C catalyst before ADT, and much superior activity to Pt/C after 10K ADT cycles.

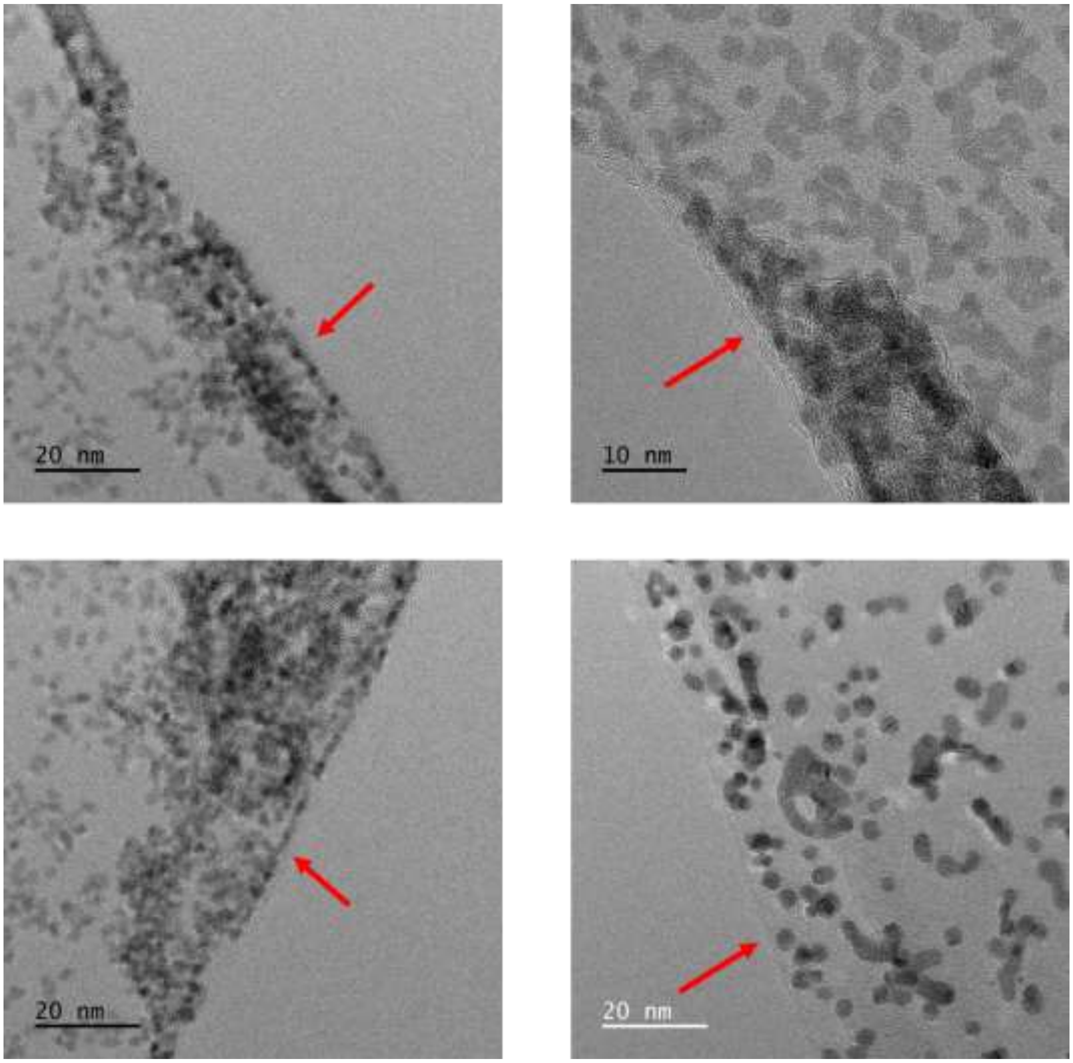

Figure S10: TEM images showing few-layers graphene thickness at the edges of GR/Pt/GR sandwich structure.

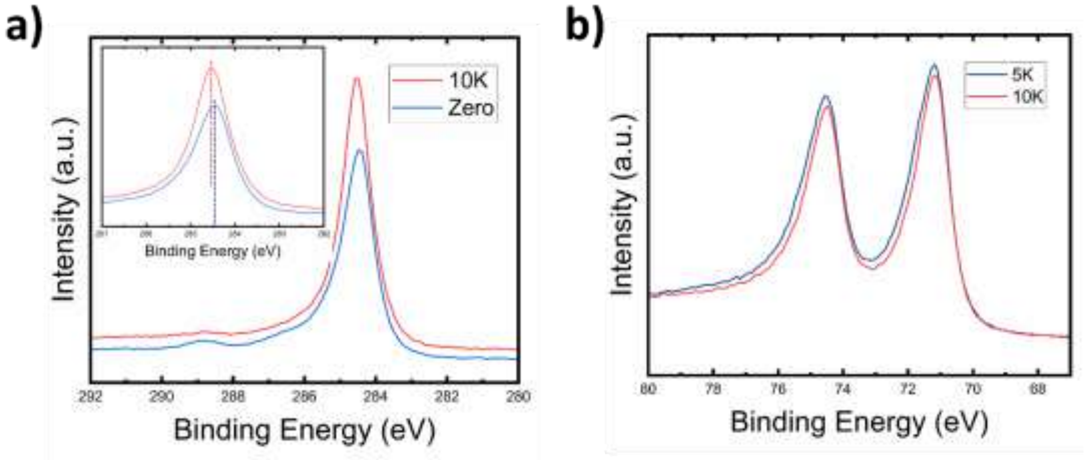

Figure S11: XPS spectra of GR/Pt/GR sandwich structure tested after different testing ADT intervals. a) C 1s spectra before ADT (blue) and after 10K cycles (red). b) Pt 4f spectra recorded after 5K and 10 K ADT cycles, blue and red respectively.

References

1. Dhingra, S., et al., *Chemical vapor deposition of graphene on large-domain ultra-flat copper.* Carbon, 2014. **69**: p. 188-193.
2. Vlassiouk, I., et al., *Large scale atmospheric pressure chemical vapor deposition of graphene.* Carbon, 2013. **54**: p. 58-67.
3. Vlassiouk, I., et al., *Role of hydrogen in chemical vapor deposition growth of large single-crystal graphene.* ACS nano, 2011. **5**(7): p. 6069-6076.
4. Li, X., et al., *Large-area graphene single crystals grown by low-pressure chemical vapor deposition of methane on copper.* Journal of the American Chemical Society, 2011. **133**(9): p. 2816-2819.
5. Li, X., et al., *Graphene films with large domain size by a two-step chemical vapor deposition process.* Nano letters, 2010. **10**(11): p. 4328-4334.
6. Abdelhafiz, A., et al., *Epitaxial and atomically thin graphene- metal hybrid catalyst films: the dual role of graphene as the support and the chemically- transparent protective cap.* Energy & Environmental Science, 2018. **11**(6): p. 1610-1616.
7. Abdelhafiz, A., et al., *Layer-by-Layer Evolution of Structure, Strain, and Activity for the Oxygen Evolution Reaction in Graphene-Templated Pt Mono layers.* Acs Applied Materials & Interfaces, 2015. **7**(11): p. 6180-6188.
8. Vitale, A., et al., *Sandwiched Graphene Interdiffusion Barrier for Preserving Au@ Pt Atomically Thin Core@ Shell Structure and the Resulting Oxygen Reduction Reaction Catalytic Activity.* ACS applied materials & interfaces, 2018. **11**(1): p. 1026-1032.
